# Double Heteroatom Reconfigured Polar Catalytic Surface Powers High-Performance Lithium–Sulfur Batteries

**DOI:** 10.3390/ma15165674

**Published:** 2022-08-18

**Authors:** Zeyuan Shi, Bo Gao, Rui Cai, Lei Wang, Wentao Liu, Zhuo Chen

**Affiliations:** 1 Key Laboratory for Ecological Metallurgy of Multimetallic Mineral, Ministry of Education, Northeastern University, Shenyang 110819, China; 2 School of Metallurgy, Northeastern University, Shenyang 110819, China

**Keywords:** lithium–sulfur batteries, heteroatoms, polarization, surface reconfiguration

## Abstract

The modification of apolar carbon materials by heteroatom doping is an effective method that can effectively improve the surface polarity of carbon materials. In the main body of the lithium–sulfur battery cathode, the structural properties of the carbon material itself with porous structure and large specific surface area provide sufficient space for sulfur accommodation and mitigate the bulk effect of the sulfur cathode (79%). The polarized surface of the reconstructed carbon material possesses strong adsorption effect on LiPs, which mitigates the notorious “shuttle effect.” In this paper, the surface structure of the Ketjen black cathode body was reconstructed by B and N double heteroatoms to polarize it. The modified polarized Ketjen black improves the adsorption and anchoring ability of LiPs during the reaction and accelerates their kinetic conversion, while its own uniformly distributed small mesopores and oversized BET structural properties are beneficial to mitigate the bulk effect of sulfur cathodes. Lithium–sulfur batteries using B and N modified cathodes have an initial discharge capacity of 1344.49 mAh/g at 0.1 C and excellent cycling stability at 0.5 C (381.4 mAh/g after 100 cycles).

## 1. Introduction

Lithium–sulfur batteries are considered one of the alternatives for next-generation energy-storage products due to their high theoretical specific capacity (1675 mAh/g), energy density (2600 Wh/kg), abundant natural reserves of sulfur monomers, and environmental friendliness. However, the sulfur cathode of Li-S batteries still faces some challenges, such as the insulation of monolithic sulfur, the sulfur bulk effect (79%) during cycling, and the “shuttle effect” of lithium polysulfides (LiPs). These problems have seriously hindered the practical application of Li-S batteries [1,2,3,4].

To solve these problems, researchers have compounded sulfur with different types of carbon materials to form active cathodes [5,6]. It has been demonstrated that the use of these carriers can effectively seal polysulfides and improve the utilization of sulfur cathodes by taking advantage of their structural characteristics, such as porosity and high specific surface area [7]. However, the impact of apolar carbon materials on improving the performance of lithium–sulfur batteries is limited. Researchers have compounded sulfur with modified carbon materials to reconfigure the anode surface by introducing multiple active adsorption sites (e.g., N, P, S, and Se heteroatoms) [8,9]. The polarized carbon material enhances the binding ability to polysulfides, slowing down the capacity degradation caused by the “shuttle effect” [10,11,12]. Fortunately, the introduction of different heteroatoms not only increases the number of active sites on the surface but also acts as a catalyst for the conversion of sulfur species during the cycling process [13,14].

Ketjen black materials have good electronic conductivity and their application in cathode carriers for lithium–sulfur batteries have proven to be effective [15]. The introduction of B and N heteroatom-doped [16] Ketjen black as cathode sulfur bodies for lithium–sulfur batteries can reconstruct the surface of Ketjen black [17], add a large number of surface active adsorption sites [18,19], and enhance the binding ability with polysulfides. The role of B and N is also reflected in the catalytic acceleration of the redox process of sulfur species under the good electronic conductivity of the Ketjen black body [20,21,22,23,24]. Therefore, the introduction of multiheteroatom-functionalized modified materials into the cathode carrier of lithium–sulfur batteries is an effective strategy to slow down the “shuttle effect.”

## 2. Materials and Methods

### 2.1. Preparation of B, N Double-Doped Ketjen Black

Boric acid, urea, and Ketjen black were weighed according to the mass ratio of 1:3:1, dissolved in deionized water, and stirred to make them evenly dispersed in water, then divided into three parts, transferred into the polytetrafluoroethylene liner of the hydrothermal reactor, and placed in the hydrothermal reaction in a blast-drying oven at temperatures of 200 °C, 175 °C and 150 °C for 8 h to obtain the B and N double-doped Ketjen black.

### 2.2. Battery Positive Electrode Preparation

Take sulfur and Ketjen black mass ratio 7:3, mix well and melt load at 155 °C for 12 h. Weigh the active substance, conductive agent, binder 7:2:1 mass ratio, add a small amount of NMP and stir for 6–8 h to make a slurry, coat on aluminum foil, dry under vacuum at 60 °C for 12 h, cut into 10 mm × 10 mm size round positive electrode by cutting machine.

### 2.3. Structural Characterization

X-ray diffraction (XRD) maps of modified Ketjen black were obtained using an X-ray diffractometer (Shimadzu 7000, Daojin, Japan). Scanning electron microscopy (SEM) from Zeiss, Germany was used to obtaining the morphological and structural information of the material. Raman signals between 800 and 3000 cm^−1^ were obtained using a LabRAM HR800 Raman spectrometer (Horiba Jobin Yvon, Paris, France). The specific surface area and pore size distribution of the modified Ketjen black were analyzed using an APSP 2460 N_2_ adsorption/desorption analyzer (Micromeritics, Norcross, GA, USA). X-ray photoelectron spectroscopy (XPS) was obtained using a K-Alpha spectrometer (Thermo Fisher Scientific, Waltham, MA, USA).

### 2.4. Electrochemical Testing

A button cell (type 2032) was used to test the electrochemical properties of modified Ketjen black. The 10 mm × 10 mm circular cathode sheets were assembled as button cells in a glove box, and the electrolyte used consisted of 1.0 M LiTFSI-DOL: DME with a volume ratio of 1:1 and 2.0% LiNO_3_ (dodochemicals.com). Lithium foil (Φ15.6 mm, 0.45 mm, China Energy Lithium Co, Tianjin, China) was used as the negative electrode of the half cell. Coin-cell constant-current charge/discharge tests were performed using a voltage window range of 1.7–2.8 V. Electrochemical AC impedance spectroscopy (EIS) tests, and cyclic voltammetry (CV) tests were performed on the cells using a CHI 760E electrochemical workstation (Ch Instruments, Bee Cave, TX, USA) with a frequency range of 0.1–10^6^ Hz on a Sunway high-performance battery test instrument. To verify the catalytic effect of modified Ketjen black on the conversion of LiPs, Li_2_S nucleation dissolution tests were performed. The prepared BN/KB/S and KB/S are used as cathodes. The lithium foil is used as the anode. The cathode solution consists of 18 µL of 0.25 M Li2S8 and 1.0 m LiTFSI in a tetramethylene glycol dimethyl ether solution. In the case of the anode electrolyte, it consists of 18 µL of 1.0 M LiTFSI DOL: DME = 1:1 and 2.0% LiNO3. To convert the polysulfide Li2Sx (x = 6, 8) to Li2S4, the following procedure was used. The cell was held at a constant potential of 2.04 V until the current dropped to 0.01 mA. In the Li2S dissolution test, the cell was first discharged at 0.10 mA to 1.70 V and then at a constant current of 0.01 mA to 1.70 V to completely reduce the S species to solid Li2S. Subsequently, the cell was charged at a constant potential of 2.32 V to convert Li2S to polysulfide until the charging current fell below 0.01 mA.

## 3. Results and Discussion

**Figure 1:**
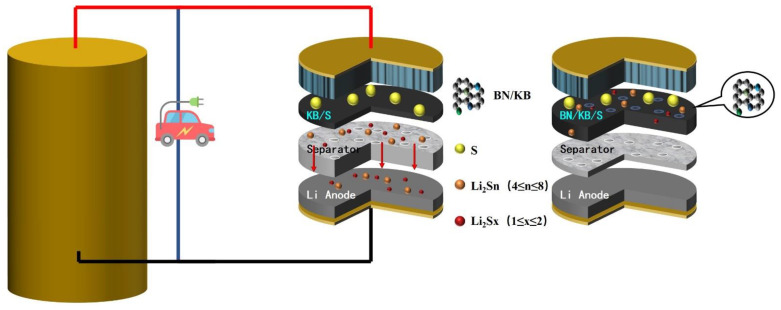
Schematic diagram of lithium–sulfur battery structure and modified structure.

The internal structure of a lithium–sulfur battery is composed of the positive electrode, diaphragm, and negative electrode. A lithium–sulfur battery with KB as the sulfur carrier lacks the anchoring effect on LiPs, resulting in uneven deposition onto the lithium cathode surface through the porous diaphragm, causing severe dendritic phenomena (Figure 1). In contrast, the doped BN/KB material not only serves as a sulfur carrier, but the introduction of B and N also reconfigures the surface of KB material [18,19], making it polarized and improving its ability to bind LiPs [25], thus reducing the capacity degradation caused by the “shuttle effect.” In addition, the BN/KB material can effectively counteract the bulk effect of sulfur (79%) due to its structural properties (large BET) [15].

**Figure 2:**
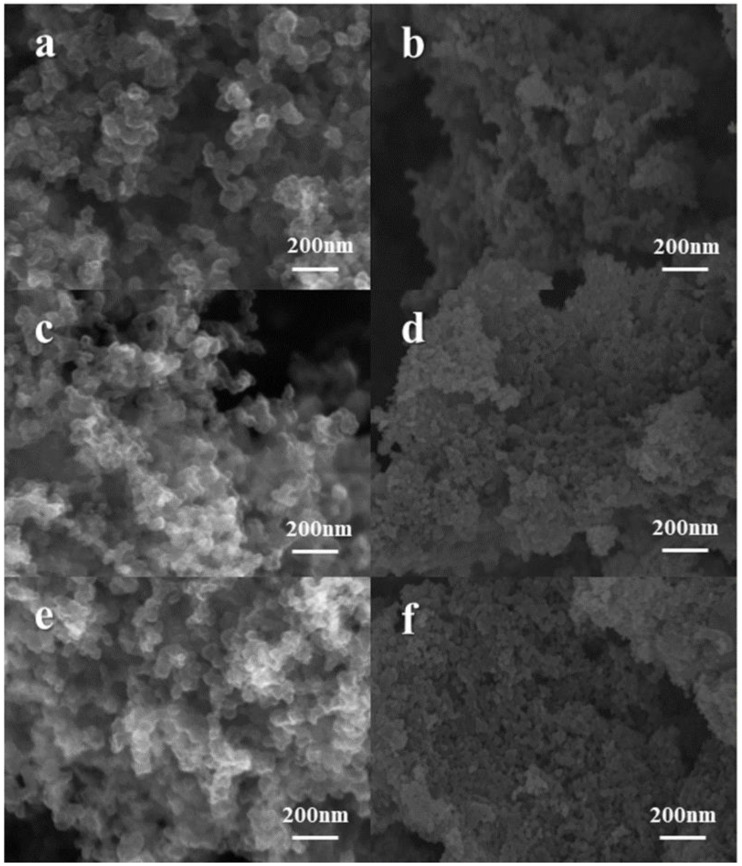
SEM morphology of BN/KB materials at different temperatures and morphology after loading S (**a**) 150 °C BN/KB (**b**) 150 °C BN/KB (**c**) 175 °C BN/KB (**d**) 175 °C BN/KB (**e**) 200 °C BN/KB (**f**) after load S BN/KB (**f**) 200 °C BN/KB after load S.

A total of six specimens of the Ketjen black carrier material modified by three temperature gradients of B/N double doping at 150 °C, 175 °C, and 200 °C, and its corresponding cathode material after S loading was subjected to SEM characterization to study the surface morphology of each sample. From Figure 2a,c,e, it can be observed that with the increase in temperature during the doping modification process, the carbon nanoparticles on the surface of Ketjen black material accumulate more closely, and the color of the material is also lighter, which indicates that the carbon particles of Ketjen black form a more stable contact form and the number of internal micropores of Ketjen black material is greatly increased, which can be more favorable for sulfur loading. In addition, the modified Ketjen black has a lighter and thinner organization in the material stack, which can be better adapted to alleviate the volume changes during the charging and discharging of lithium–sulfur batteries [26]. Combined with the electron micrographs of modified Ketjen black after sulfur loading at three temperature gradients of Figure 2b,d,e, it can be observed that in Figure 2b the bonding is looser than in Figure 2d,f, and it can be judged that the sulfur loading is worse than the other two. Compared with KB/S, the BN/KB modified at different temperatures were better than the blank control group (Figure S1).

It is concluded that after the modification operation of Ketjen black carrier doping under three different temperature conditions, the modified Ketjen black carrier material has the best structural improvement at 200 °C. Meanwhile, the modified sample with sulfur loading can completely and uniformly accommodate the active material S8 in a large number of microporosity inside the material, and there is no obvious sulfur particle clustering on the surface at the nanoscale [27,28].

**Figure 3:**
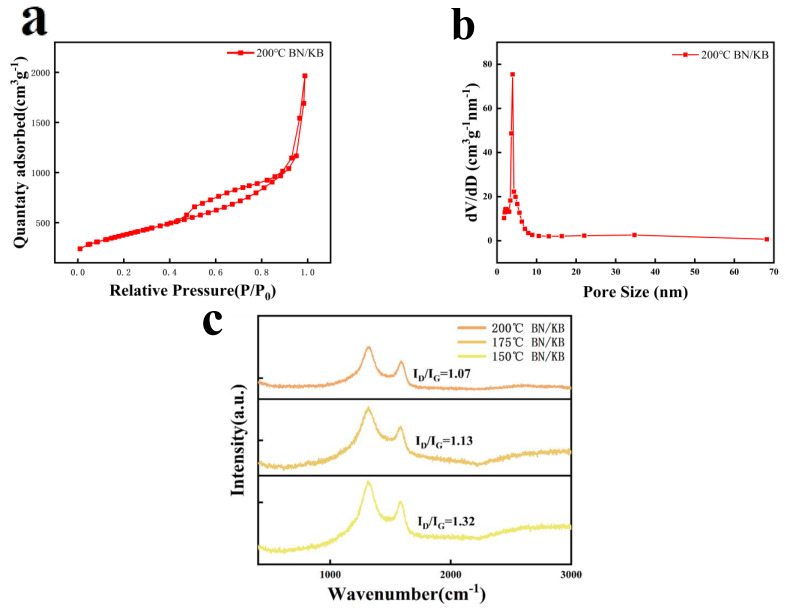
Specific surface area (**a**) and pore-size distribution (**b**) of BN/KB. (**c**) Raman spectrum of BN/KB material at 200 °C, 175 °C, and 150 °C.

The physical properties of the B and N double-doped modified Ketjen black carrier material were analyzed, and the best results were tested by BET for the double-doped modified Ketjen black material at 200 °C. The specific surface area of the modified Ketjen black carrier material at 200 °C was 1365.3758 m^2^/g and the average pore size was 8.6541 nm (Figure 3b). The adsorption curve is a typical type IV adsorption curve (Figure 3a), and also exhibits an H3-type hysteresis loop adsorption isotherm curve, which generally occurs in the flake-particle material. It can be concluded that the microporosity in the tested material is formed by the stacking slit of flake particles, and its pores do not exhibit saturation in the higher relative pressure region of adsorption. Based on the BET analysis, it can be further concluded that the structure of the Ketjen black carrier material is more inclined to lamellar thin-layer stacking after the double-doping modification at 200 °C. The carbon particles of its composition are stacked thinner and form a more solid contact structure, and the porosity is also increased [28,29]. Fortunately, the higher specific surface area and richer pore structure can better facilitate electrolyte infiltration into the cathode material and block the diffusion of intermediate LiPs with its excellent structural characteristics [15].

The modified materials were further analyzed by Raman spectroscopy, and two characteristic peaks were observed at 1280 cm^−1^ (D) and 1580 cm^−1^ (G) for the modified Ketjen black materials at 150, 175, and 200 °C (Figure 3c). The D peak morphology is caused by carbon atoms with incomplete graphitic microcrystals, unsaturated edges, and many structural defects in the material, reflecting the sp^3^-type non-graphitized disordered carbon structure contained in the activated Ketjen black, and the G peak indicates the stretching vibration of atoms within the sp^2^ hybridized carbon crystal plane. I_D_/I_G_ = 1.32 after 150 °C, I_D_/I_G_ = 1.13 at 175 °C, and I_D_/I_G_ = 1.05 at 200 °C were obtained, indicating that the non-graphitized structural defects within the Ketjen black material tend to decrease with increasing temperature of the modification process within a certain temperature range. The blank control group had the largest I_D_/I_G_ of 1.41 (Figure S2). After the modification process of B/N double doping at different temperatures, part of the σ-bonds are redistributed with the participation of B atoms to produce a new linkage form of π-bonds, which reconstructs this part of the structure into a planar-like sp^2^-type graphitized structure, thus improving to some extent the properties of Ketjen black as an active material for a lithium–sulfur battery cathode. The properties of Ketjen black as a carrier for lithium–sulfur batteries have been improved to some extent, demonstrating the microscopic repair ability of element B for carbon structures [30].

**Figure 4:**
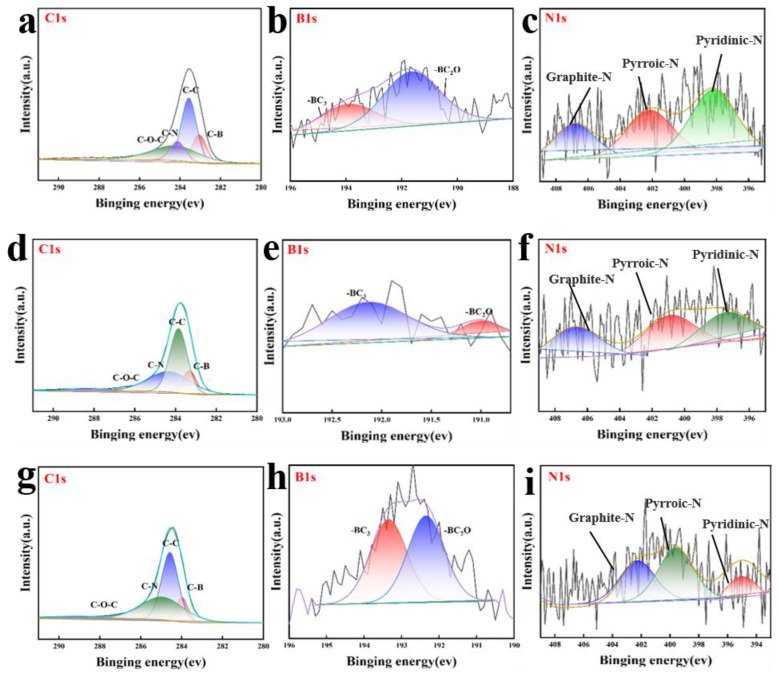
XPS fine spectra of BN/KB materials at different temperatures. (**a**–**c**) are the fine spectra of C1s, B1s, and O1s at 150 °C. (**d**–**f**) are the fine spectra of C1s, B1s, and O1s at 175 °C. (**g**–**i**) are the fine spectra of C1s, B1s, and O1s at 175 °C.

The two fitted peaks were −BC_3_ (193.5 eV) and −BC_2_O (192.1 eV) in the high-resolution spectrum of B 1 s [30] (Figure 4c,e,h). The introduced B elements can repair the defects in the original structure and maintain the structural stability of the material, which provides a guarantee for good electrochemical cycling stability. Meanwhile, the doped reconstructed polarized surface helps to improve the binding and anchoring of LiPs. From the high-resolution spectra of C 1s, the peak located at 284.6 eV is mainly from graphite-like carbon (C = C) with sp^2^ hybridization after B-structure repair, while the peaks located at 283.9 eV and 285.2 eV are attributed to C–B and C–N, and the peak at 286.5 eV is the O–C functional group of sp^3^ carbon. The peak of the C–B group at 283.9 eV indicates the successful doping of the B atom, and the peak of the C–N group at 285.2 eV indicates the successful doping of the N atom. (Figure 4a,d,g). The N doping forms pyridine N, pyrrole N, and graphite N structures that form a stronger anchoring and binding to the LiPs [19,25,31] (Figure 4c,f,i), thus reducing the unstable cycling ability due to shuttle effects. In the blank control group KB/S, since there is no heteroatom orbital structure formed by B and N (Figure S3), it does not show good performance in the subsequent electrochemical performance tests. The uniform peak height corresponding to B can be observed in the fine spectrum of B 1 s at 200 °C. It is demonstrated that the doping of B causes a relative decrease in the degree of material defects as the temperature increases under the temperature gradients to 150 °C, 175 °C, and 200 °C, which exactly corresponds to the lowest-value I_D_/I_G_ of BN/KB material at 200 °C in Raman spectra, fully reflecting the superiority of B and N double heteroatom doping [32].

**Figure 5:**
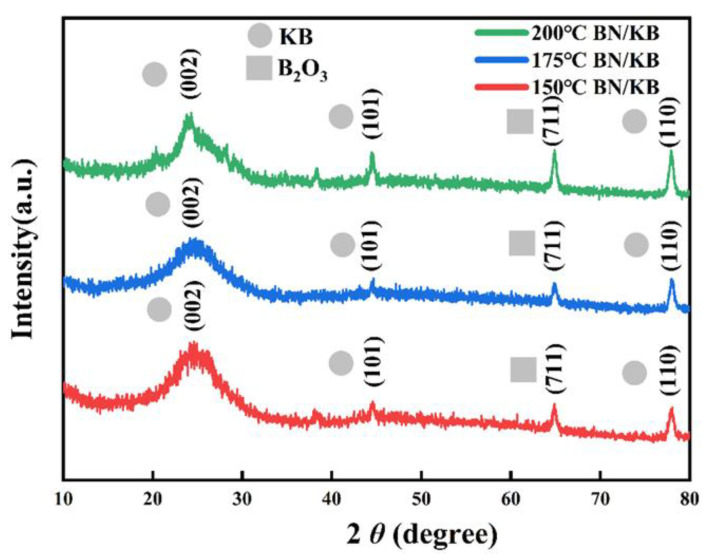
XRD of BN/KB material at 200 °C, 175 °C, 150 °C.

As shown in Figure 5, all three modified Ketjen black materials show three characteristic peaks at 2θ values of ~26°, ~43°, and ~78°, corresponding to the coincidence of (002), (101), and (110) crystallographic planes of Ketjen black material. The appearance of the characteristic peak at the value of 2θ of ~65° coincides with the (711) crystal plane of B_2_O_3_, indicating that part of the B element exists in the form of boron oxide crystals (hexagonal crystalline boron oxide (α-B_2_O_3_)). The reason for this peak is that boric acid decomposes and dehydrates to produce B_2_O_3_ at 150 °C. As the temperature of the B/N double-doping modification process increases, the better the crystallinity. In the blank control KB, due to the lack of the effect of temperature and heteroatoms, it showed a wider amorphous peak shape (Figure S4).The doping of a certain amount of B_2_O_3_ can enhance the performance of the microporosity of the material, while its good chemical anchoring effect on LiPs, the intermediate product of lithium–sulfur batteries, and the presence of a small amount does not affect the electronic conductivity of the material [33].

**Figure 6:**
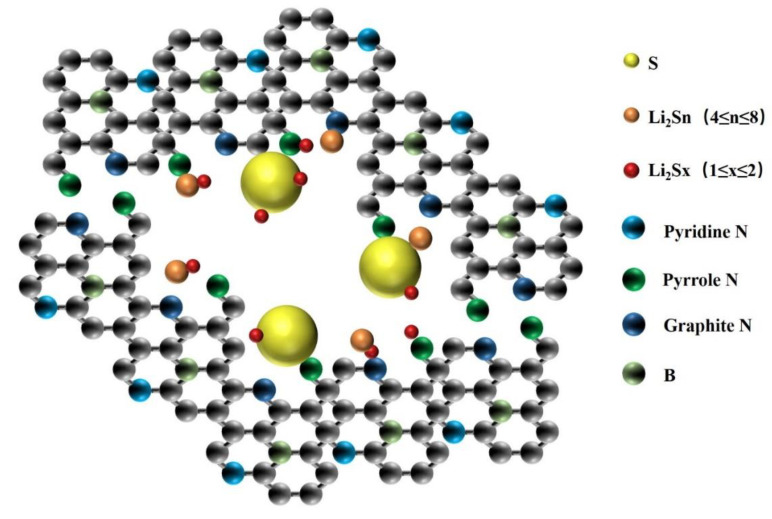
Illustrations of the anchoring effect of BN-reconfigured polarized surfaces on polysulfide species.

The introduction of B and N double heteroatom-modified Ketjen black material (BN/KB), based on the highly symmetric structure of the initial KB material surface, the weak interaction between apolar and polar molecules, results in the weak interaction between KB and LiPs, causing a serious “shuttle effect.” In contrast, BN/KB materials not only have the −BC3 and −BC2O structures formed by the introduction of B to enhance the anchoring of polysulfide molecules [30] but also have the pyridine N, pyrrole N, and graphite N structures formed by the KB materials [18,19,31], which also exhibit the promotion of the kinetic conversion of LiPs species based on the good electronic conductivity of KB materials (Figure 6) [13,34]. Thus, the cycling stability of LiPs batteries was improved.

**Figure 7:**
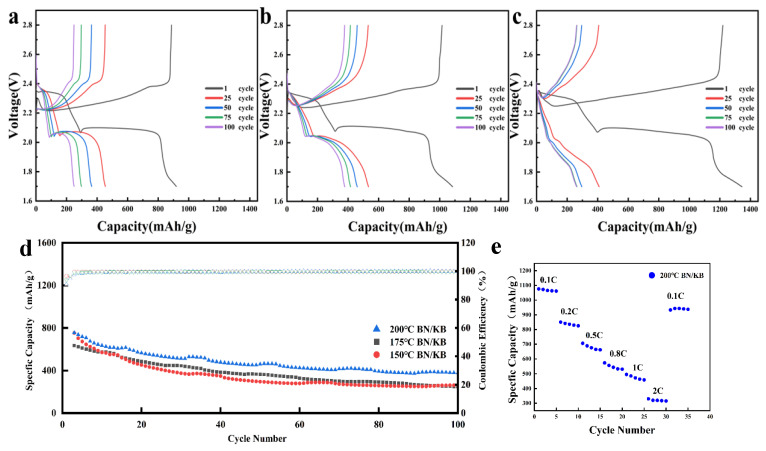
Charge/discharge plateau curves (**a**–**c**) and specific capacity cycling curves (**d**) for BN/KB at 200 °C, 175 °C and 150 °C, rate performance (**e**).

The cyclic charge/discharge plateau curves showed that the lithium–sulfur batteries prepared by BN/KB had the highest first-cycle discharge-specific capacity of 1344.49 mAh/g at 175 °C (Figure 7c), and the modified samples had the best capacity retention at 200 °C (Figure 7b). Moreover, the lithium–sulfur batteries prepared by BN/KB all showed a typical double-charge/discharge plateau (Figure 7a) (2.4–2.1 V for the first stage and 2.1–1.7 V for the second stage). The typical double-discharge plateau was still maintained after 100 charge/discharge cycles, which proves that BN/KB material as the cathode carrier has better electrode structural stability and good electrode kinetic performance, and thus has a more excellent long-cycle performance [35] (Figure 7d). In the blank control KB, due to the lack of B and N polarized catalytic surfaces, the initial specific capacity of the battery is not high, and the cycle decay is severe (Figure S5). Among them, BN/KB at 200 °C still showed good specific capacity retention after 100 cycles at 0.5C (Figure 7d). In the rate test, the BN/KB cell showed excellent rate performance at 0.1C, 0.2C, 0.5C, 0.8C, 1C, 2C, and 0.1C with 1075.7,841.3,706.3,573.9,485.6,320.1,933.2 mAh/g (Figure 7e).

**Figure 8:**
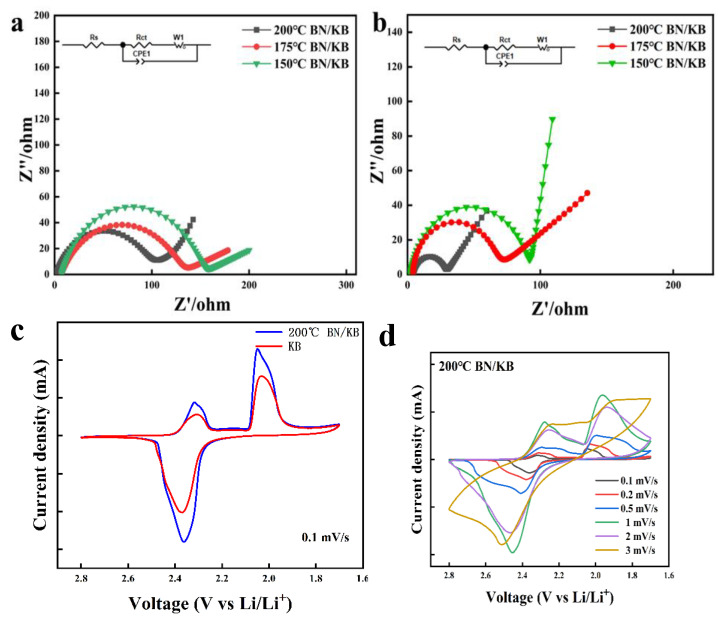
Nyquist plots of BN/KB cells before and after cycling at 200 °C, 175 °C, and 150 °C and the corresponding equivalent circuits (**a**,**b**). CV of BN/KB vs. KB (**c**) and CVs of BN/KB at 200 °C at different sweep speeds (**d**).

The effect of different temperature conditions on the electronic conductivity of the material was further investigated. The half circles in the high-frequency region of the Nyquist plot represent the interfacial resistance (Rct) (Figure 8). The values of BN/KB Rct before cycling, at 150 °C, 175 °C, and 200 °C are 159.7 Ω, 138.34 Ω, and 109.68 Ω (Figure 8a). This proves that in a certain temperature range, the material interface electronic conductivity obtains a certain degree of enhancement with the increase in temperature. After 100 cycles at 0.5 C, the Rct decreased to different degrees: 100 Ω, 75.3 Ω, and 28.76 Ω. In the blank control KB, due to the lack of graphitic N to improve the electronic conductance of the material, the Nyquist plot changed from 201.5 Ω initially to 170.1 Ω after 100 cycles (Figure S6). The reason for this phenomenon may be that the electronic conductivity of the electrode is influenced by the microstructure of the electrode material, and the conductive paths remain stable and numerous after long cycles [36,37,38].

The electrochemical performance of BN/KB and KB cells was compared at a sweep rate of 0.1 mV/s between 1.7 V and 2.8 V. The electrochemical performance of BN/KB cells was examined at different sweep rates. The typical two different cathode peaks, located at 2.35 V and 2.01 V (Figure 8c), are the reduction of S to LiPs (Li_2_Sx, 4 ≤ x ≤ 8) during discharge, and the reduction of soluble polysulfide to insoluble Li_2_S_2_/Li_2_S. The 2.38 V peak is the oxidation of Li_2_S_2_/Li_2_S to S during charging. Peak separation was observed with an increasing sweep rate at 200 °C in the BN/KB cell, the two cathode peaks are negatively shifted by 0.07 and 0.09 V, while the anode peak is positively shifted by 0.12 V (Figure 8d) [39]. Thus, the polar surface formed by BN/KB and the higher redox peak of the BN/KB cell can effectively reduce the electrochemical polarization and enhance the kinetic process [40], providing assurance of long cycle performance [41,42].

**Figure 9:**
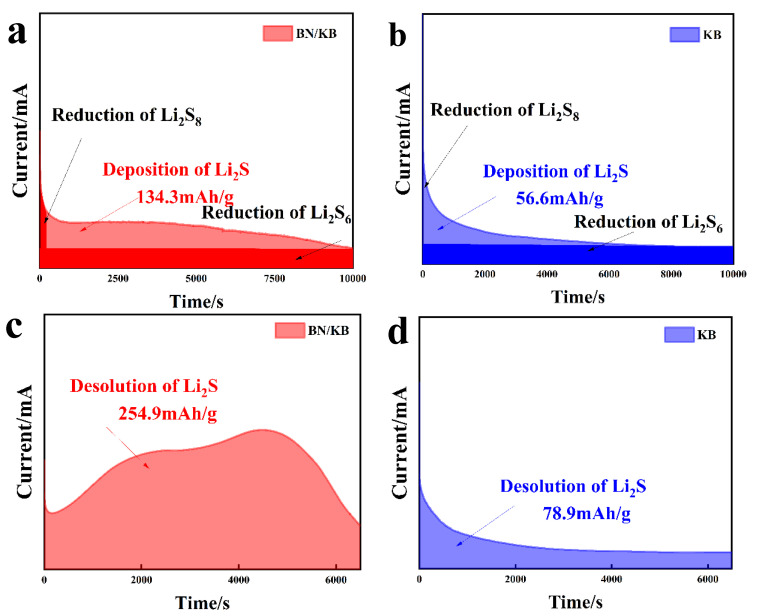
(**a**,**b**) Potentiostatic discharge profiles at 2.04 V on BN/KB and KB electrodes with Li_2_S_8_ catholyte. (**c**,**d**) Potentiostatic charge profiles at 2.32 V to evaluate the dissolution kinetics of Li_2_S.

To further evaluate the catalytic effect of the electrode materials on the reversible reaction between polysulfide and Li_2_S, potentiostatic nucleation and dissolution experiments were carried out [39]. The current density of Li_2_S at 2.05 V on BN/KB electrodes is larger than that on KB. According to Faraday’s law, the precipitation capacity of the BN/KB electrode is 134.3 mAh/g, which is higher than 56.6 mAh/g for the KB electrode (Figure 9a,b). Meanwhile, the constant potential Li_2_S dissolution experiments (Figure 9c,d) showed that the current density and dissolution capacity of BN/KB electrode (254.9 mAh/g) was much higher than that of KB (78.9 mAh/g). These results provide further evidence that the introduction of double heteroatoms in BN/KB enhances the deposition and kinetic dissolution of Li_2_S [34]. Overall, the presence of B and N heteroatoms not only reconfigures the material surface and provides sufficient polysulfide interaction sites [18] but also provides excellent active sites for the reversible phase-transition process of Li_2_S [31,43].

## 4. Conclusions

In conclusion, the double heteroatomic B and N doped Ketjen black material is an effective sulfur host material for lithium–sulfur batteries. The combination of XPS, SEM, Raman, XRD, and BET confirms that the synthesized BN/KB materials possess good physical structural properties. The B and N heteroatoms not only reconfigure the polar surface of the materials to anchor more soluble polysulfides but also provide enough catalytic active sites to provide sufficient redox kinetic capabilities in the interconversion of sulfur, polysulfides, and Li_2_S. As a result, the BN/KB-based sulfur cathode exhibits good electrochemical performance, showing both excellent multiplicative performance and excellent cycling stability at different current densities. In the study of BN/KB double heteroatom doping, this polar surface structure helps to achieve excellent electrochemical performance. This approach can be tried and explored not only for sulfur carriers but also for other kinds of batteries and energy-storage forms.

## Data Availability

Data available in a publicly accessible repository.

## Supplementary Materials

The following supporting information can be downloaded at: https://www.mdpi.com/article/10.3390/ma15165674/s1, Table S1 The performance comparison of this work with some other similar researches. FigureS1. SEM images of (a) Ketjenblack and (b) KB/S cathodes. Figure S2. Raman spectroscopy of Ketjenblack. Figure S3. XPS photoelectron spectroscopy of cotinine black positive carrier material (a) full spectrum of cotinine black (b) C1s fine spectrum (c) O1s fine spectrum. Figure S4. XRD of Ketjenblack. Figure S5. (a) KB/S positive cyclic curve. (b) Cycle platform curve of KB/S positive pole under different cycle numbers. Figure S6. AC impedance spectra (EIS) of Cochin black cathode cells before and after cycling (a) EIS impedance before cycling (b) EIS impedance after cycling. References [44,45,46] are cited in the supplementary materials.
