# Supplementary material for: Double Heteroatom Reconfigured Polar Catalytic Surface Powers High-Performance Lithium–Sulfur Batteries
Source: Materials (Basel). 2022 Aug 18;15(16):5674. doi: 10.3390/ma15165674 (PMC9412490; doi:10.3390/ma15165674)
Supplement: Supplementary file 1 [file materials-15-05674-s001.zip › materials-1863453-supplementary.pdf]

Supporting Information

Double heteroatom reconfigured polar catalytic surface powers high-performance lithium-sulfur batteries

Zeyuan Shi [1], Bo Gao [1,*], Rui Cai [2], Lei Wang[1]., Wentao Liu [2] and Zhuo Chen [2]

[1] Key Laboratory for Ecological Metallurgy of Multimetallic Mineral (Ministry of Education), Northeastern University, Shenyang, Liaoning Province, China, 110819
[2] Northeastern University, Shenyang, Liaoning Province, China, 110819
* Correspondence: Corresponding: gaob@smm.neu.edu.cn

Table S1. The performance comparison of this work with some other similar researches.

Coating	Cycle number	C-rate	Initial specific capacity（mAh/g）	Ref
KB/TiO_2	100	0.2C	1130.3（0.2C）	[1]
KB/Fe_3O_4	100	0.2C	1274.5（0.2C）	[2]
KB/Co	100	0.5C	1249.0（0.05C）	[3]
BN/KB	100	0.5C	1344.5（0.1C）	This work

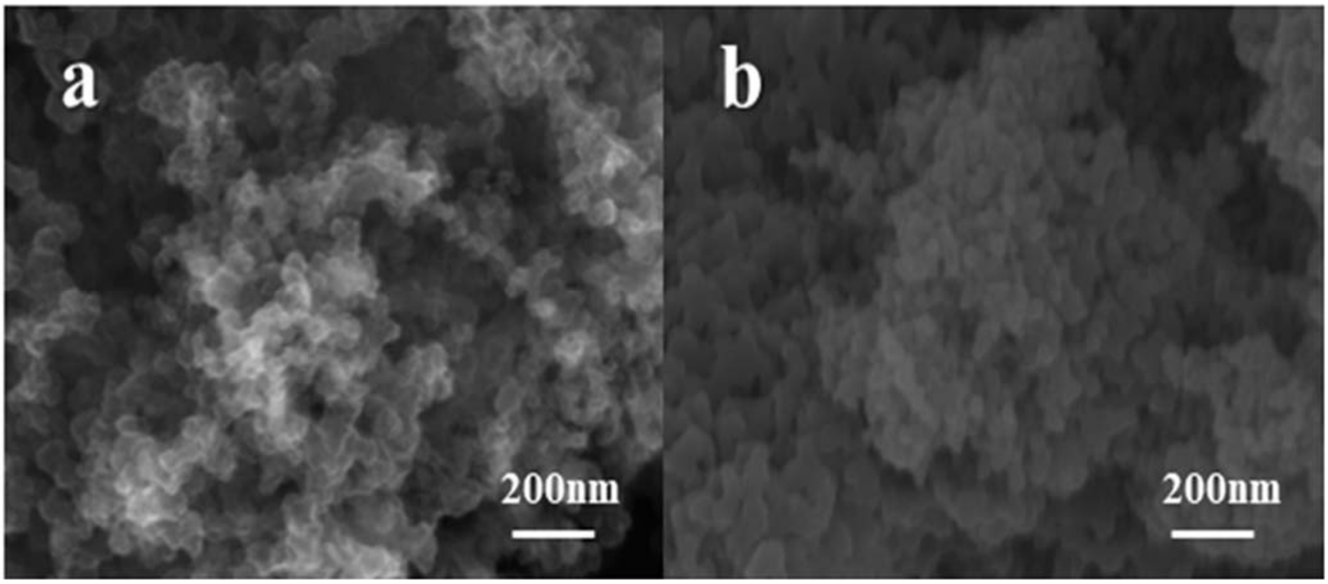

Figure S1. SEM images of (a) Ketjenblack and (b) KB/S cathodes

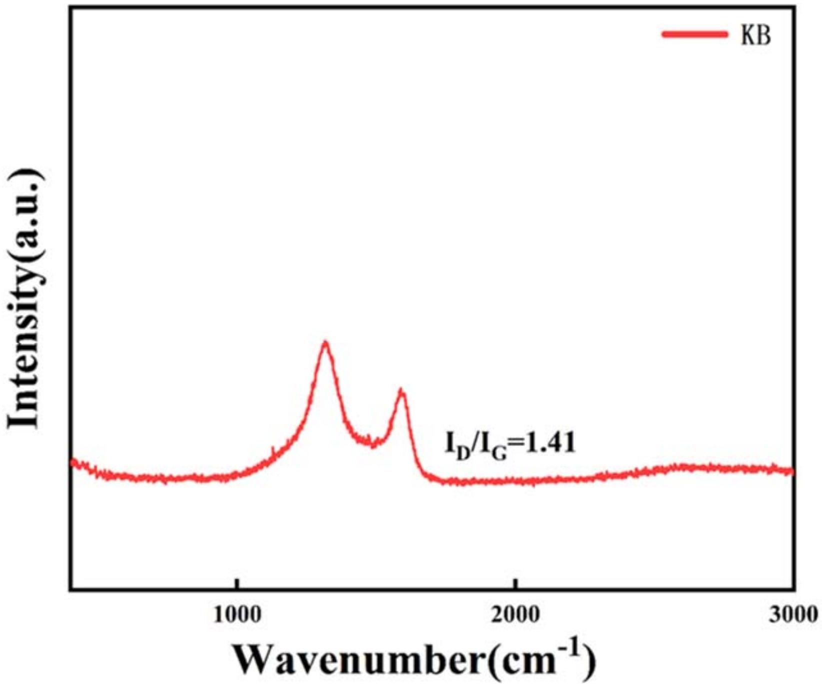

Figure S2. Raman spectroscopy of Ketjenblack

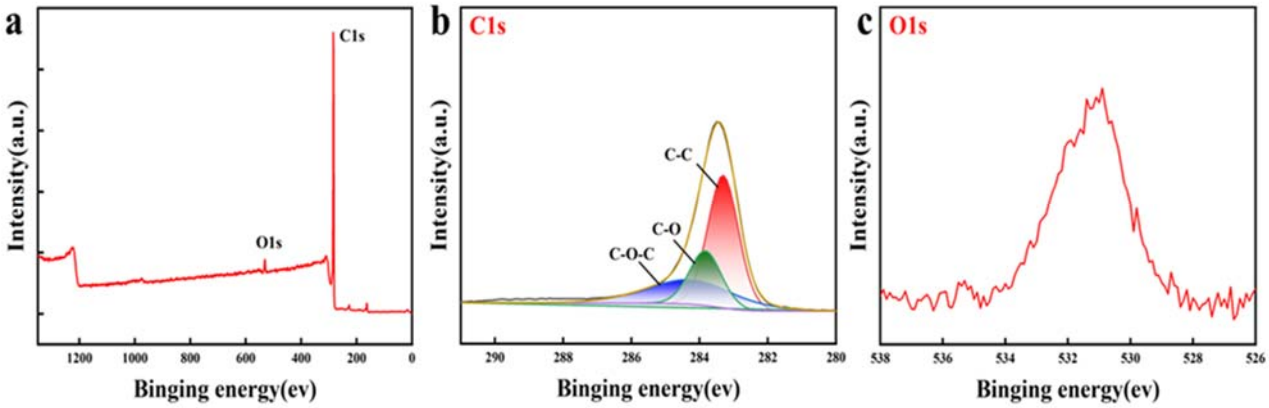

Figure S3. XPS photoelectron spectroscopy of cotinine black positive carrier material (a) full spectrum of cotinine black (b) C1s fine spectrum (c) O1s fine spectrum

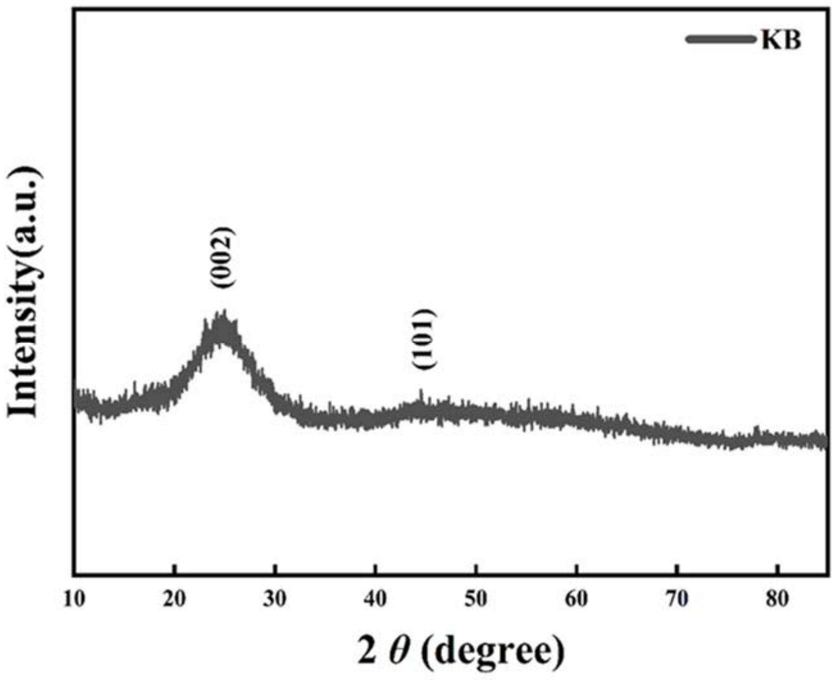

Figure S4. XRD of Ketjenblack

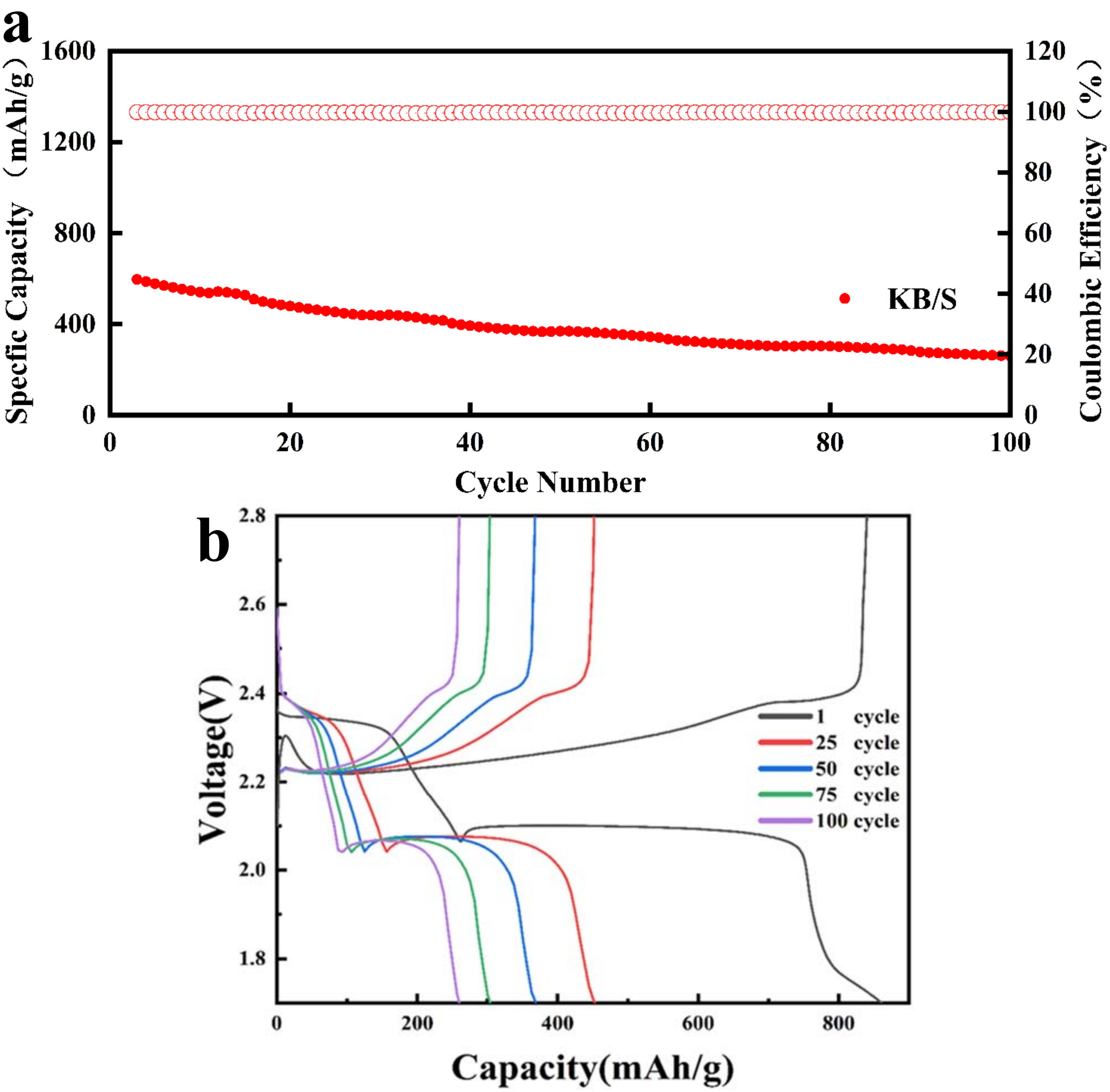

Figure S5. (a) KB/S positive cyclic curve. (b) Cycle platform curve of KB/S positive pole under different cycle numbers

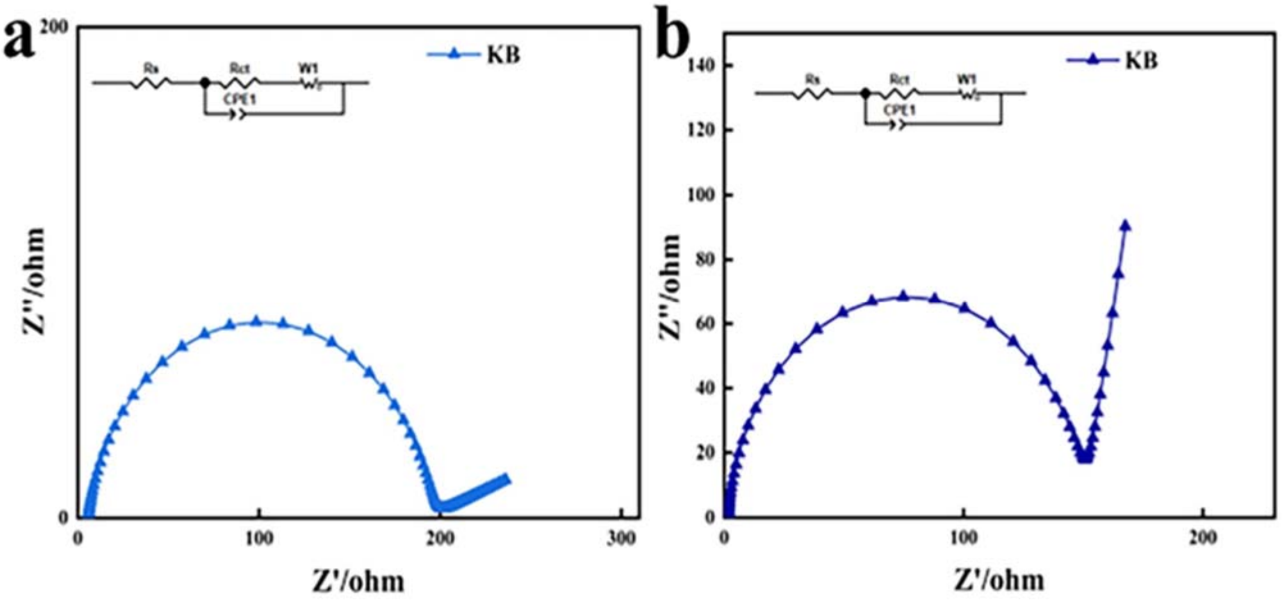

Figure S6. AC impedance spectra (EIS) of Cochin black cathode cells before and after cycling (a) EIS impedance before cycling (b) EIS impedance after cycling.

References

1. Shan, L.; Yurong, C.; Jing, Y.; Feixia, R.; Jun, W.; Babu, S.; Xin, Y.; Junkuo, G.; Juming, Y., Entrapment of polysulfides by a Ketjen Black & mesoporous TiO2 modified glass fiber separator for high performance lithium-sulfur batteries. *Journal of Alloys and Compounds* **2019,** 779, 412-419.
2. Zhu, R.; Lin, S.; Jiao, J.; Ma, D.; Cai, Z.; Hany, K.; Hamouda, T. M.; Cai, Y., Magnetic and mesoporous Fe3O4-modified glass fiber separator for high-performance lithium-sulfur battery. *Ionics* **2019,** 26, (5), 2325-2334.
3. Wang, B.; Li, T.; Qian, X.; Jin, L.; Yao, S.; Shen, X.; Qin, S., In situ growth of Co nanoparticles in Ketjen Black for enhanced electrochemical performances of lithium-sulfur battery cathode. *Journal of Solid State Electrochemistry* **2021,** 25, (5), 1579-1590.
